# Math Error Types and Correlates in Adolescents with and without Attention Deficit Hyperactivity Disorder

**DOI:** 10.3389/fpsyg.2017.01801

**Published:** 2017-10-11

**Authors:** Agnese Capodieci, Rhonda Martinussen

**Affiliations:** ^1^Department of General Psychology, University of Padova, Padova, Italy; ^2^Department of Human Development and Applied Psychology, University of Toronto, Toronto, ON, Canada

**Keywords:** ADHD, math abilities, types of errors, switching, adolescents

## Abstract

**Objective:** The aim of this study was to examine the types of errors made by youth with and without a parent-reported diagnosis of attention deficit and hyperactivity disorder (ADHD) on a math fluency task and investigate the association between error types and youths’ performance on measures of processing speed and working memory.

**Method:** Participants included 30 adolescents with ADHD and 39 typically developing peers between 14 and 17 years old matched in age and IQ. All youth completed standardized measures of math calculation and fluency as well as two tests of working memory and processing speed. Math fluency error patterns were examined.

**Results:** Adolescents with ADHD showed less proficient math fluency despite having similar math calculation scores as their peers. Group differences were also observed in error types with youth with ADHD making more switch errors than their peers.

**Conclusion:** This research has important clinical applications for the assessment and intervention on math ability in students with ADHD.

## Introduction

Attention Deficit Hyperactivity Disorder (ADHD) is a developmental disorder characterized by symptoms of inattention, impulsivity, and hyperactivity (American Psychiatric Association, 2013) which are associated with difficulties in a wide range of academic skills including mathematical computation and problem-solving, reading and language comprehension, and written expression (Bonafina et al., 2000; DuPaul et al., 2004; Hart et al., 2010; Gremillion and Martel, 2012). While academic achievement has been studied extensively in children with ADHD, less is known about academic skills in adolescents with ADHD – particularly in the domain of mathematics (Wolraich et al., 2005). Given the importance of mathematics to an individual’s future health and employment status (Reyna et al., 2009; Ritchie and Bates, 2013), this is a gap that needs to be addressed.

Math appears to be an area of challenge for a number of individuals with ADHD (Tosto et al., 2015). Tosto et al. (2015) reviewed studies investigating math ability in individuals with ADHD who were between the ages of 6 years old and adulthood. They found that 83% of the studies they reviewed reported a statistically significant negative association between ADHD symptoms and mathematical performance. Studies from Tosto’s review highlighted the presence of weaknesses in math fluency and math calculation in children and youth with ADHD. Understanding why children and youth with ADHD show weaknesses in math fluency and calculation is an important research avenue as such information may aid in developing intervention or instructional approaches for this group of students.

One way to gain insight into the nature of the challenges youth with ADHD experience with math fluency is to examine the type of errors that the students make and to assess whether they are similar or different to those made by peers. Although prior studies have explored error patterns in mathematics tasks in children with ADHD (Benedetto-Nasho and Tannock, 1999; Re et al., 2016), it is not clear whether youth with ADHD would make more and specific types of errors in comparison with their non-affected peers on a math fluency task. This is an important gap in the field as poor math fluency may constrain the choices that youth with ADHD make in secondary course selection as they may avoid courses that involve math which in turn may limit their employment options (Fourqurean et al., 1991).

Prior research on math error types and ADHD has focused on children and has identified errors that appear to be related to inattentiveness as well as working memory (Benedetto-Nasho and Tannock, 1999; Raghubar et al., 2009). For example, Benedetto-Nasho and Tannock (1999) found a deficit in math computation performance in their sample of children with ADHD, other researchers hypothesized that math errors could be the result of inattentiveness. Highly inattentive students may not carefully monitor their performance for mistakes during calculation tasks (Raghubar et al., 2009). Such errors would likely occur randomly rather than systematically and reflect an overall lack of monitoring for errors and monitoring ongoing task demands. These types of errors may also be related to problems with working memory as children may lose their place or focus while completing a basic math problem which may increase a range of error types in computation. Difficulty shifting or switching between operations is another error type that has been identified in children with math disabilities (Rourke, 1993). The shifting error only occurs when students switch from completing one type of task (e.g., addition) to another type of math operation (e.g., subtraction; Rourke, 1993). It is interesting to note that researchers studying cognitive shifting often use mathematical switch tasks to index shift-time costs on timed tasks (e.g., Plus-Minus task; St Clair-Thompson, 2011). Hence, one might expect that shift errors would be most evident on a calculation fluency task that requires frequent shifts between operations (plus, minus, multiply, and divide).

While inattentiveness and working memory (which are often correlated with each other, Martinussen and Tannock, 2006) are each associated with math errors (Bull and Scerif, 2001; Rogers et al., 2011), it is not clear if processing speed weaknesses also contribute to a specific pattern of errors during math calculation tasks. This is an interesting variable to study given that processing speed weaknesses (e.g., digit naming speed) are associated with ADHD (Shanahan et al., 2006; Inoue et al., 2008) and are related to less proficient math fluency (Bull and Johnston, 1997). It is possible that individual differences in processing speed are related only to overall productivity (how many questions are answered), but not to specific types of errors.

This study examines error types on a standardized math fluency assessment in youth with a parent-reported diagnosis of ADHD (with confirmation through parent report of current clinically significant symptoms) and youth without ADHD. Given the importance of mathematics to an individual’s future employment status (Reyna et al., 2009; Ritchie and Bates, 2013), we believe it is important to analyze the error performance of adolescents with ADHD while completing a timed task to better understand why their performance may be less accurate and/or fluent than their peers. Fluency in particular is important to examine because calculation fluency supports the development of skills such as fractions concepts (Jordan et al., 2013) which in turn predict more advanced math skills (Bailey et al., 2012; Booth et al., 2014).

In the present study, we used the Woodcock Johnson Test of Achievement- Third Edition (WJ-III; Woodcock et al., 2001) Math Fluency subtest to examine error types in single digit operations in youth with ADHD under speeded conditions. This test requires respondents to quickly solve single digit calculation problems that are presented in a mixed format. Hence, this test gave us the opportunity to examine errors that occurred as a result of a need to shift between operation sets (e.g., addition to subtraction) as well as examine other types of errors (e.g., basic operation error). As all calculation problems in the WJ-III are single digit, we were unable to examine errors when completing double digit operations.

We expected that youth with ADHD would exhibit weakness in math fluency relative to their typically developing peers given the association between ADHD and math fluency reported in previous studies (Ackerman et al., 1986; Biederman et al., 2005; Gray et al., 2015). We also predicted the youth with ADHD to make more switch math errors than their peers. Switch errors require attention as well as the ability to shift set and thus we anticipated that this type of error would be most prevalent in youth with ADHD given the association between executive function weaknesses (e.g., working memory, shifting, inhibitory control) and ADHD status in children, youth, and adults (Willcutt et al., 2005; Rohlf et al., 2012; Bueno et al., 2014; Holmes et al., 2014). Finally, we also expected that working memory and processing speed would be associated with a greater number of errors that are indicative of poor cognitive control and attention (e.g., switching errors, incorrect operation errors) (Baddeley et al., 2001; Draheim et al., 2016).

Summarizing, the objectives of the present study was to analyze the types of errors made from adolescents with ADHD in a math fluency task compared to their peers and the association between the types of errors and working memory and processing speed.

## Materials and Methods

### Participants

Data were collected from 109 participants between the ages of 14 and 17 who took part in a larger study conducted by one of the authors and colleagues (2015) examining academic performance and text comprehension in adolescent with and without ADHD. The inclusion criteria for ADHD group were: (1) At least one clinically significant score (*T* ≥ 70) on the Diagnostic and Statistical Manual of Mental Disorders-IV inattention (α = 0.93), hyperactivity-impulsivity (α = 0.92), or global index subscales of the Conners Third Edition Parent Rating Scales (Conners, 2008); (2) A parent-report of a diagnosis of ADHD from a psychologist or a physician; (3) an estimated intelligence quotient (IQ) ≥ 70 based on the Wechsler Abbreviated Scale of Intelligence (WASI; Wechsler, 1999) non-verbal reasoning and vocabulary subtests. The inclusion criteria for the control group were: (1) No parent-reported diagnosis of ADHD and no clinically significant symptoms of ADHD as indexed by the DSM-IV Inattention and Hyperactivity and Global Index subscales of the Conners Third Edition Parent Rating Scales (*T* ≤ 65); (2) An estimated intelligence quotient (IQ) ≥ 70 based on the Wechsler Abbreviated Scale of Intelligence (WASI; Wechsler, 1999) non-verbal reasoning and vocabulary subtests. Adolescents were also excluded if they had received a prior genetic or neurological disorder diagnosis (e.g., autism spectrum or Tourette’s syndrome) according to parent report, but other diagnoses (e.g., conduct, mood, or learning disorders) were permitted if the adolescent met all other criteria.

We focused in this study on youth who were not perceived to have a concurrent math disability in basic computation skills according to the performance on the WJ-III Math Calculation subtest. However, as there is no ‘gold standard’ to define mathematical ability (Tosto et al., 2015), we decided to draw on recent research examining adolescent outcomes of children with ADHD where “academic competence” was defined as scoring at or above the 16th percentile on standardized reading and math achievement measures from the WJ-III (Woodcock et al., 2001; Lee et al., 2008). As a result, we decided to only include students whose standard score on the WJ-III Math Calculation subtest was at or above the 16th percentile (SS ≥ 85). According to these various criteria, seven adolescents who did not have a parent-reported diagnosis of ADHD were excluded from the sample. Six participants without a diagnosis who met at least one clinical score on the Conners Third Edition Parent Scale were also excluded from the sample. Four participants who had not completed the math tests and 7 who scored less than 85 on the WJ-III Math Calculation subtest were excluded from the present sample. Finally, 16 participants who had not completed the WASI were excluded.

Therefore, our sample included 30 students (21 male and 9 female) with ADHD and 39 (15 male and 24 female) TD peers who were matched on age, IQ and parent education level (mother, father or the mean of both). Group demographic characteristics are presented in **Table 1**. Parent education was gathered from a parent demographic survey with 1 = No schooling; 2 = Some elementary; 3 = Completed elementary; 4 = Some secondary; 5 = Completed secondary; 6 = Some college; 7 = Completed college program; 8 = Some university; 9 = Completed undergraduate; 10 = Master’s degree; 12 = Doctoral degree.

**Table 1:** Demographic characteristics by group.

	ADHD (*N* = 30) Mean (*SD*)	TD (*N* = 39) Mean (*SD*)	*F*(1,67)
Age	15.49 (0.97)	15.39 (0.88)	0.64
Conners 3rd Parent DSM-IN T-score	79.83 (15.65)	49.85 (6.67)	116.13***
Conners 3rd Parent DSM-HI T-score	81.40 (16.33)	49.78 (6.93)	119.00***
Conners 3rd Parent learning problems T-score	68.53 (15.92)	49.69 (8.01)	41.21***
Conners 3rd Parent EFs problems T-score	74.37 (10.31)	49.97 (6.80)	139.60***
WASI estimated IQ	109.03 (13.05)	112.67 (8.65)	1.93
Parent education level	8.82 (2.16)	8.11 (2.17)	1.80


**N*, number; ADHD, attention deficit hyperactivity disorder; TD, typically developing adolescent; DSM, diagnostic and statistical manual of mental disorder (DSM-IV TR); IN, inattentive; HI, hyperactivity-impulsivity; prob., problems; EFs, executive functions; WASI, Wechsler Abbreviated Scale of Intelligence; IQ, intelligence quotient; SD, standard deviation. ^∗^*p* < 0.05, ^∗∗^*p* < 0.01, ^∗∗∗^*p* < 0.001.*

### Procedures

This study was carried out in accordance with the recommendations of the second author’s institutional research ethics board with written informed consent from all subjects’ parents/guardians. All subjects gave written informed consent in accordance with the Declaration of Helsinki. An intake screen was first completed by telephone with the interested parents/guardians to determine each youth’s eligibility for the study. Parents/guardians of eligible youth were provided with an information and consent letter to inform them of the study. All parents of the participating youth in this study provided their written consent for the youth to participate in the study. In addition, at the start of each visit to the lab, the research assistant explained the study to each youth and acquired their verbal assent to take part in the study. All youth were reminded that it was their choice to take part in the study and then could stop taking part at any time. Research assistants worked individually for about 5 h with each participant and all participants were provided with breaks when needed. During the assessment, the adolescents completed standardized achievement tests and a battery of self-report measures relating to beliefs and attributions for behavior that were part of a larger study.

### Measures

#### Conners Third Edition-Parent and Adolescent (Conners 3)

The Conners Third Edition- Parent and Adolescent (Conners 3) is a reliable and well-validated diagnostic instrument for ADHD, including adolescent self-report and parent versions (Conners, 2008). The Learning Problems and Executive Functioning subscale scores were used in the present study. The Learning Problems subscale (Parent α = 0.90; Adolescent α = 0.84) represents an indicator of perceived academic competence (i.e., the lower the subscale score, the higher the academic competence). High scores on the Executive Functioning (Parent α = 0.92) subscale indicate behavioral problems associated with executive functions (EFs) deficits and acts as an informant-based measure of broader EFs impairments. EFs problems was added to provide an indication of the level of parent reported EF difficulties in both groups. For each item, respondents were asked to evaluate on a 4-point scale (0 = never, rarely; 3 = really true, 4 = very often) the extent the item was true in the past month. Adolescents completed a self-report version; parents completed a parent version. Raw scores were converted to age- and gender-specific standardized *T*-scores.

#### Wechsler Abbreviated Scale of Intelligence

The Wechsler Abbreviates Scale of Intelligence (WASI; Wechsler, 1999) is a brief, standardized, and well-normed test of verbal and non-verbal intelligence. The Matrix Reasoning and Vocabulary subscales were administered to adolescents to provide a screening measure of general cognitive ability (IQ). The WASI Vocabulary and Matrix Reasoning subtests result in a *T*-score with a mean of 50 and a standard deviation (*SD*) of 15. The reliability coefficients reported in the manual for Vocabulary in children aged 14 to 16 the range is 0.90 to 0.93 and for Matrix Reasoning they ranged from 0.86 to 0.91.

#### Woodcock–Johnson Tests of Achievement – Third Edition

Math abilities were assessed through two tests of WJ-III: Math Calculations and Math Fluency (Woodcock et al., 2001). Math Calculations measures the ability to perform mathematical computations. The items required the students to perform basic operations, as well as some geometric, trigonometric, logarithmic, and calculus operations. The calculations involved negative numbers, percent, decimals, fractions, and whole numbers. Math Fluency measures the ability to solve simple single-digit addition, subtraction, and multiplication facts quickly and in random order so switching from one operation to another was common. The students had to solve these simple arithmetic problems within 3 min. The WJ-III Math Calculations subscale is reported in the manual to have a median reliability of α = 0.85 in the age 5–19 range. The Math Fluency Subscale has a median reliability of α = 0.89 in the age range. Each test has a raw score that is converted to an age based standard score.

#### Test of Memory and Learning – Second Edition

The Test of Memory and Learning – Second Edition is a well-validated, standardized assessment of memory ability (Reynolds and Voress, 2007). From this comprehensive test, the adolescents completed the Digit Forward and Digit Backward subtests. Digit Forward and Digit Backward were used to measure verbal working memory. In the Digit Forward task, students were presented a series of digits (e.g., 5, 9, and 6) and they are asked to repeat the series back to the examiner immediately. The Digit Backward task is similar but the participants need to reverse the order of the numbers when providing the series back to the examiner. The Digit Forward and Digit Backward subtests’ raw scores were converted to age adjusted scaled scores which have a mean of 10 and SD of 3. According to the manual for Digit Forward the coefficient α = 0.97 for ages 14–16. For Digit Backward the range is 0.97–0.98.

#### Comprehensive Test of Phonological Processing

We administered the Rapid Digit and Color Naming subtests from the Comprehensive Test of Phonological Processing (CTOPP; Wagner et al., 1999) which is a well-validated, standardized assessment of processing speed abilities and switching. Adolescents were asked to name aloud as fast as possible the names of numbers (Digit Naming) and colors (Color Naming). Not all students of the sample could complete these subtests as the measures were added after some data had been collected in the study. We converted the raw scores to age adjusted scaled scores which have a mean of 10 and *SD* of 3. The coefficient alpha for Digit Naming for youth aged 14–16 range from 0.85 to 0.93 whereas the Color Naming reliability ranges from 0.81 to 0.86.

#### Types of Errors in Math Fluency

We decided to analyze four types of errors in Math Fluency. First, we reviewed previous studies examining math errors (Benedetto-Nasho and Tannock, 1999; Raghubar et al., 2009) and from these studies we decided to code the Math Fluency subtest for four types of math errors:

(a) Incorrect operation errors: this type of error is when the participant gave a response that could be correct if the sign of the operation was different (e.g., 5 + 2 = 3 or 6 - 2 = 8).
(b) Basic errors: the type of error is a basic error in operations and seems to show a lack of understanding of the operation (e.g., 5 × 3 = 16).
(c) Switch errors: this type of perseverative error occurred when the student’s error on the math problem was incorrect and it showed the persistence of using the operation from the previous question on the test (e.g., 4 + 2 = 6; 5 - 3 = 8).
(d) Zero errors: This type of error is a specific type of basic error in which the operation contained a 0 (e.g., 5 × 0 = 1).

The numbers of error for each category were calculated as the percentage of error on the total numbers of solved operations in the 3-min time limit (Benedetto-Nasho and Tannock, 1999). For example, if a student solved correctly 82 operations and made 1 incorrect operation and 2 basic errors, he completed in total 85 operations. So, we calculated that he made 1.18% of incorrect operation errors and 2.35% of basic errors.

#### Interrater Reliability (IRR)

We calculated the interrater reliability of the math errors types. We asked a second coder with clinical training in administering the WJ-III Math Fluency measure to rate a random sample of 15% of the total sample of protocols. IRR was assessed using a two-way mixed, absolute agreement ICC (Intraclass Correlation Coefficient; McGraw and Wong, 1996). The resulting ICC was in the excellent range, ICC = 1.00 (Cicchetti, 1994) for all types of errors, indicating that coders had a high degree of agreement and suggesting that types of errors were rated similarly across coders.

#### Statistical Analyses

We compared adolescents with ADHD and TD students on math measures using a univariate analysis of variance (ANOVA). The differences between the two groups in the type of errors in Math Fluency were analyzed using a Mann–Whitney *U* test of variance involving non-parametric variables that do not have a normal distribution. In fact, many participants made no errors, or they made only one to two errors of a particular kind. For an effect size, Grissom and Kim (2012) have suggested that for two-group independent samples design, one can determine an effect size dividing the Mann–Whitney *U* statistic by the product of the two sample sizes. For all the analyses, we controlled for the gender effect. We only reported gender differences when there were significant gender effects in the analyses. We used Spearman correlations to examine the associations between math error types and the processing speed tasks and used logistic regression to determine whether one or both of the cognitive variables (working memory, processing speed) predicted error rates controlling for ADHD group status.

## Results

**Table 2** presents the results of the first set of analyses on the Math Calculation and Fluency subtests as well as the math error types. There was no statistically significant difference between the ADHD and TD peers on the WJ-III Math Calculation subtest. However, youth with ADHD did score significantly lower than their TD peers on the WJ-III Math Fluency subtest (*F* = 21.02, *p* < 0.001). There was a significant difference between the two groups on the two processing speed tests, Digit Naming (*F* = 6.22, *p* = 0.016) and Color Naming (*F* = 7.94, *p* = 0.007) with youth with ADHD showing slower processing speed relative to their peers on both tasks. There were no statistically significant differences in working memory performance between the two groups. There were no statistically significant differences in the math error types although we found that the effect size for the number of switch errors was moderate in size (*U* = 467, *p* = 0.063, η^2^ = 0.40) with the ADHD group making more switch errors that the typically developing comparison group. Interestingly, the opposite was found for the basic errors and the incorrect operation errors with the comparison group making more of these types of errors relative to the youth with ADHD. We analyzed the frequencies of students who made the distinct types of errors in the two groups as well. We found that 12 (31%) TD students compared to 5 (17%) youth with ADHD made incorrect operations errors, 16 (41%) TD adolescents in comparison with 8 (27%) youth with ADHD made basic errors and 7 (18%) TD students compared to 11 (37%) youth with ADHD made switch errors. Only 4 (10%) TD adolescents made zero errors.

**Table 2:** Differences between adolescents with ADHD and TD on math performance, EFs and types of errors in Math Fluency.

	ADHD (*N* = 30) Mean (*SD*)	TD (*N* = 39) Mean (*SD*)	*F*(1,67)	Effect size $\eta_{p}^{2}$
Math Calculation SS	107.37 (10.49)	112.67 (12.86)	3.37	0.05
Math Fluency SS	87.73 (12.03)	101.62 (11.34)	21.02***	0.24
Digit Forward SS	12.03 (13.87)	9.95 (2.42)	0.85	0.11
Digit Backward SS	11.97 (9.95)	10.87 (2.67)	0.43	0.01

	**ADHD (*N* = 28) Mean (SD)**	**TD (*N* = 26) Mean (SD)**	***F*(1,52)**	**$\eta_{p}^{2}$**

Rapid Digit Naming SS	9.04 (2.10)	10.39 (1.86)	6.22*	0.11
Rapid Color Naming SS	8.68 (2.09)	10.23 (1.95)	7.94**	0.13

	**Mean (*SD*)**	**Mean (*SD*)**	***U* (67)**	**η^2^**

Incorrect operation errors	0.24 (0.58)	0.37 (0.63)	510	0.44
Basic errors	0.31 (0.54)	0.57 (0.87)	506	0.43
Switch errors	0.60 (0.88)	0.28 (0.73)	467	0.40


**N*, number; ADHD, attention deficit hyperactivity disorder; TD, typically developing adolescent; SD, standard deviation; SS, standard score. ^∗^*p* < 0.05, ^∗∗^*p* < 0.01, ^∗∗∗^*p* < 0.001.*

### Association between Processing Speed and Math Error Types

When we analyzed the Spearman correlations between the types of errors and the two processing speed measures we found a significant negative correlation between the switch errors and the Color Naming test (*r* = -0.335, *p* = 0.013). Considering the correlations between the types of errors and the two working memory measures we found a negative correlation between the switch errors and the Digit Backward test (*r* = -0.213). Therefore, participants with a high number of switch errors tended to have lower scores (slower naming speed) on the Color Naming and Digit Backward subtest. We decided not to analyze the zero errors due to the low frequency of this kind of errors. The correlations was analyzed even separately for the two groups but being similar we decided to consider the correlation in the whole sample.

As the distributions of the errors were not normal, with many students making no errors, or many making only one to two errors of a particular variety, we decided to create new dichotomous variable for switch errors. One group was comprised of youth who made no switch errors vs. those who made at least one error (% of errors < 1 = 0, % of errors > 1 = 1). A logistic regression was then conducted to predict switch error type using the Digit Backward and Color Naming subtests (standard scores, SS) as predictors with ADHD status entered first. We only included these subtests as it were the only shown to be correlated with switch errors. Different variables were introduces in different blocks. Preliminary analyses indicated that ADHD status when entered alone was a significant predictor of being a member of the switch error group (odds ratio = 3.91, *p* = 0.010). Youth with ADHD were at increased risk relative to their peers of being members of the switch errors subgroup. However, as shown in **Table 3**, none of the variables (ADHD status, Color Naming, Digits Backward) when entered together were significant unique predictors of membership in the switching errors subgroup. However, youth with better Color Naming tended to be at less risk (odds ratio = 0.69, *p* = 0.082) which is consistent with the correlation between Color Naming and switch errors. Also, when entered alone, Color Naming was a predictor of group membership with youth with better Color Naming at lower risk of being a member of the switch errors group (odds ratio = 0.77, *p* = 0.031). Considering the interaction between ADHD status and Color naming it was not significant (odds ratio = 0.28, *p* = 0.108).

**Table 3:** Logistic regression to predict the switch errors in Math Fluency in students with Math Calculation SS > 85.

Variable	*B*	*SE*	Wald	*df*	*p*	OR
Presence of ADHD (yes vs. no)	0.59	0.81	0.52	1	0.470	1.80
Color Naming SS	-0.37	0.21	2.96	1	0.082	0.69
Digit Backward SS	-0.07	0.16	0.19	1	0.671	0.94


*Different variables were introduced in different blocks; SE, standard error; *df*, degree of freedom; OR, odds ratio.*

## Discussion

The present study provided an analysis of the type of errors that adolescents with ADHD made in a single-digit timed math fluency task, an understudied aspect of mathematical abilities in adolescents with ADHD (Tosto et al., 2015). Importantly, we restricted our sample to youth who did not exhibit marked math calculation difficulties. Given that there is no gold standard to define mathematical ability (see Murphy et al., 2007) we decided only included students with a SS in Math Calculation greater or equal to 85 when conducting the comparisons of the two groups in math abilities, EFs and types of errors. It is possible that had we selected a more stringent cut-off our findings may have changed.

Our first set of analyses revealed that there were no statistically significant differences in Math Calculation between the two groups. This result could be partially due to the restriction of the sample (students with SS in Math Calculation greater or equal to 85) but the same effect could be expected for the Math Fluency. Instead, consistent with prior research (Tosto et al., 2015), there was a significant difference for Math Fluency with youth with ADHD exhibiting less fluent math calculation skills than their peers. Concerning the types of errors, in this case we found that adolescents with ADHD made more switch errors than their peers although the difference was not statistically significant. The effect size, however, was moderate in size indicating that more studies are needed with larger samples to better understand error types in youth with ADHD. In contrast, youth in the comparison group made more basic and zero errors (both procedural errors but in the second case the operation contained a zero). In these analyses, the magnitude of the difference was moderate (Cohen, 1988). TD children likely showed a higher number of basic and zero errors due to the fact that they attempted to complete a great numbers of operations in the 3 min time at disposal [TD mean of total operations done 105 (25) vs. 80 (25) of adolescent with ADHD].

Concerning processing speed, we found that students with ADHD had a lower performance in Color Naming relative to the comparison group. This finding is consistent with previous literature that found an impairment in processing speed in children with ADHD (Rucklidge and Tannock, 2002; Shanahan et al., 2006) and in real-life measures (Lawrence et al., 2004). In contrast, there were no statistically significant differences on the Digits Backward subtest which was our measure of working memory. Given that working memory tends to be lower in individuals with ADHD relative to peers (Willcutt et al., 2005), this finding is somewhat surprising. It is possible that by excluding youth with ADHD with math difficulties we also reduced the number of youth with ADHD with less proficient working memory – this would be an interesting question to address in future research. Alternatively, our measure of working memory may not have placed sufficient load on working memory in a sample of youth with ADHD.

When we conducted the logistic regression to determine if the switch errors in Math Fluency were predicted by the two cognitive measures, we found that Color Naming was not a unique predictor when concurrently entered along with ADHD status and working memory. However, when entered on its own, the results of the logistic regression revealed that it was a significant contributor to classification and better Color Naming was associated with lower odds of a youth being a member of the switch errors group.

In summary, even though the adolescents with ADHD in our sample demonstrated average performance in a math calculation task, they performed significantly lower on the math fluency task compared to their non-affected peers. The math fluency measure we used requires youth not only to use their math abilities but also switch from one operation to another in a 3-min time limit. It is likely that the poorer performance on the math measure is in part due to slow fluency (as indicated by the Color Naming speed task) but also from the nature of the task as it required careful monitoring of problem type and the ability to quickly switch operation types. Previous research suggests that students with ADHD exhibit problems when completing tasks where they must inhibit irrelevant task information when switching from one task to another (Kramer et al., 2001), so in this case to inhibit the preceding operation.

Another important variable is time. In the Math Fluency task students were only permitted 3 min to complete as much as possible of the task. Previous research examining timed tasks (Lewandowski et al., 2007), has shown that with additional time (1.5 times the time), children with ADHD were able to reach the performance level of the control group at standard time. This finding suggests that the required switching process, together with the time limit is likely having a marked impact on the math fluency performance of students with ADHD. This perspective seems to be supported by our findings of switch errors with very few basic or zero errors in adolescents with ADHD and from their poorer performance in one or both test of processing speed, which capture the ability to perform simple cognitive tasks quickly and fluently over a sustained period of time.

This study has some important from a clinical point of view inasmuch as we are interested in acquiring knowledge of “actual” math fluency in students with ADHD. It would be interesting in future research to compare the performance of timed tasks in blocks (all similar operations within a block) vs. mixed. Similar to research with individuals with ADHD on task switching (e.g., Cepeda et al., 2000), it is possible that the switch costs incurred by the nature of the task affect speed of responding as well as accuracy.

Although this research analyzed an understudied and interesting topic concerning computation error types during a math fluency task in adolescents with ADHD, there are some limitations that needed to be considered. First, our sample was small and we included only one measure of math fluency and only few measures of EFs and processing speed. It would be important to replicate our findings with a more diverse sample (perhaps children and youth) using different subgroups (e.g., math LD plus ADHD, ADHD alone). In addition, it would be interesting to compare the performance on math fluency tasks that vary in switching demands to better understand the nature of the math fluency weaknesses in children and youth with ADHD. From a clinical standpoint, this research was useful as it suggests that in some cases students with ADHD could have a lower performance on tasks assessing fluency in comparison with their peers as a result of the nature of the task (mixed blocks vs. single operation blocks). This finding suggests that from a practical perspective, it might be important for educators to consider using math fluency tasks that do not require switching between operations to better understand if the issue is fluency or the executive control needed to switch sets rapidly.

Finally, given the importance of math to future life success, it is important that more research addresses mathematics in youth with ADHD. More research is needed in this field to understand which processes (e.g., switching, inhibition, and speed), compromise the performance of adolescents with ADHD. Furthermore, multi-digit arithmetic operation should be investigated in this range of age to analyze if the pattern of errors remains the same or change with the complexity of the task and with or without the time and the switching request.

## Ethics Statement

The study from which this data was obtained was approved for ethical clearance by the University of Toronto’s Research Ethics Committee which follows the Tri-Council Policy Statement: Ethical Conduct for Research Involving Humans (2010) (http://www.research.utoronto.ca/faculty-and-staff/research-ethics-and-protections/humans-in-research/). Parents/guardians were asked to provide their consent for their child’s participation and all youth were asked to provide their assent for participation. We ensured that all assent information for the youth was presented in a way that enhanced comprehension and we prompted youth to ask questions for clarification several times during the assent process.

## Author Contributions

AC and RM contributed to the design and implementation of the research. AC contributed to the analysis of the results and to the writing of the manuscript. RM contributed to drafting the article and to the critical revision of the article.

## Conflict of Interest Statement

The authors declare that the research was conducted in the absence of any commercial or financial relationships that could be construed as a potential conflict of interest. The reviewer MCPF and handling Editor declared their shared affiliation.
